# Developing a faster way to identify biocontamination in the air of controlled environment rooms with HEPA filters: airborne particle counting

**DOI:** 10.1038/s41598-020-59367-8

**Published:** 2020-02-13

**Authors:** Angels Figuerola-Tejerina, Ana Hernández-Aceituno, Guadalupe Alemán-Vega, César Orille-García, Miguel Ruiz-Álvarez, Helena Sandoval-Insausti

**Affiliations:** 1 Service of Preventive Medicine, Hospital Universitario de La Princesa, Madrid, Spain; 2 Department of Preventive Medicine and Public Health. School of Medicine, Universidad Autónoma de Madrid-IdiPaz; and CIBERESP (CIBER of Epidemiology and Public Health), Madrid, Spain; 3 Department of Nutrition, Harvard T.H. Chan School of Public Health, Boston, MA USA

**Keywords:** Environmental monitoring, Risk factors

## Abstract

Our aim was to assess whether airborne particle counting is an immediate indicator of biocontamination in controlled environment rooms with HEPA filters in a hospital. A prospective study was carried out in a tertiary care hospital between 2016 and 2018. The study was divided in two periods and the measurements were performed in different controlled environment rooms with HEPA filters. The Environmental Biosafety Criterion (EBC) was defined as the absence of fungal and bacterial contamination. In the training period, the area under the ROC curve (aROC) of airborne particle counting and EBC was calculated for each particle size as well as the cut-off points that optimize the combination of sensitivity and specificity in the association between them. aROC is created by plotting sensitivity against 1-specificity. In the testing period, the cut-off points previously selected were validated. 328 measurements were carried out in the training period and 301 in the testing period. In the training period, an association was found between airborne particle counting and EBC. An aROC = 0.760, 95% Confidence Interval (95% CI) 0.695–0.825 was observed for 0.3 µm particles; an aROC = 0.797 (95% CI 0.734–0.860) for 0.5 µm particles; and an aROC = 0.751 (95% CI 0.673–0.829) for 5 µm particles. The cut-off points that optimized the combination of sensitivity and specificity were 9.0 × 10^3^ for 0.3 µm particles, 3.6 × 10^3^ particles for 0.5 µm, and 3.2 × 10^2^ particles for 5 µm. In the testing period, the previous cut-off points were validated. We conclude that airborne particle counting is a useful, immediate, and preliminary measure to identify the presence of biocontamination in controlled environment rooms with HEPA filters.

## Introduction

The hospital environment is a potential source of infection through air, water, and contaminated surfaces. So, it is essential to have an environmental biosafety system as a part of the patient biosafety program in each hospital.

The infection risk associated with the air in a hospital depends on different risk factors, such as the concentration of infectious particles in the air, exposure time, and the patient’s immune system.

The infections caused by opportunistic filamentous fungi such as *Mucor, Rhizopus*, and *Scedosporium*, have risen in frequency over the last decades. However, these infections remain less frequent than those related to *Aspergillus*, which could be more virulent and more difficult to deal with due to their resistance mechanisms to drugs^1^. The presence of environmental fungal contamination establishes the hygiene status of the controlled environment rooms. Furthermore, the airborne level of bacteria in the controlled environment rooms is another way to evaluate the hygiene status of these rooms. A controlled environment room is classified as a clean room when the air samples revel less than 100 Colony-Forming Units per m^3^ (CFU/m^3^), and acceptable when the room is between 100 and 200 CFU/m^3^ ^2^. A fungal contamination and/or an airborne level of bacteria superior to 200 CFU/m^3^ in a controlled environment room compels the hospital to suspend the activity in the room, inspect the air-conditioning system, and conduct a cleaning and terminal disinfection of all the surfaces. The usual activity cannot be resumed until new environmental biosafety verification is confirmed with the identification of no environmental fungal contamination and the detection of an airborne level of bacteria less than 200 CFU/m^3^. This process includes new air samplings of the room and their corresponding microbiological testing.

The definitive microbiological results of the air samplings require a minimum of 2 days for bacteria and 5 days for specific fungi^3–5^. Thus, if biocontamination is present, prompt corrective actions needed to be carried out and followed by new air samplings of controlled environment rooms. Usually, all the process implies closing the corresponding rooms for a week until the definitive results of the new microbiological measurements are available.

The Center for Disease Control and Prevention (CDC) published in 2003 the *Guide to infection control related to the hospital environment*^6^, where the scientific evidence of each published recommendation made so far was verified. One of these measures is to verify the effectiveness of the air-conditioning systems (filtration, air renewal, and pressurization) to guarantee an optimal performance in the removal of all kind of airborne particles.

Nowadays, in Spain, there is a set of regulations in which the design of the biosafety systems is based^2,6–8^. The UNE 171340:2012 regulation, *Validation and evaluation of controlled environment rooms in hospitals*, offers an excellent tool to design and check the biosafety systems and biocontamination control.

The regular or preventive verification of biocontamination in the air of the hospital controlled environment rooms consists of the inspection of the hygiene status of the rooms, the temperature, the relative humidity measurement, and the collection of samples to ascertain the presence and the counting of bacteria and fungi^9–13^.

The airborne particles samples are used to classify the risk areas^14^. The particles with a size between 0.3 μm and 10 μm are useful to establish the clean status of the air and to classify the rooms according to the International Organization for Standardization (ISO) guidelines. According to the UNE 171340:2012 regulation, the 0.3 μm airborne particle counting is used to verify the effectiveness of the High Efficiency Particulate Air (HEPA) filters. These particles indicate whether the HEPA filters are installed correctly, without drains, and free from any damage. The airborne particle counting in a room can be obtained after 5 minutes of testing.

There is the possibility of using the airborne particle counting as a prompt but provisional indicator of biocontamination in the controlled environment rooms with HEPA filters, with the aim of providing a good real time indicator for decision making, and as a method for detecting rapid variations in air quality that could go undetected by periodic air microbiological samplings.

Therefore, the aim of this study is to assess the correlation between the technique of airborne particle counting (0.3 μm, 0.5 μm, and 5 μm particle sizes), and microbiological air samplings (CFU concentration of bacteria and fungi per m^3^ of air sampling), obtained simultaneously in different controlled environment rooms with HEPA filters in a tertiary care hospital.

## Methods

Prospective study carried out in a tertiary care hospital between 2016 and 2018. The study was divided in two periods: training and testing periods. The aim of the measurements carried out during the testing period was to validate the results obtained during the training period. The duration of the training period was 2 semesters (the last semester of 2016 and the last semester of 2017) and the duration of the testing period was 1 semester (the first semester of 2018). The measurements were carried out by trained staff in a systematic way, while being supervised by a senior doctor. The measurements were performed in different controlled environment rooms with HEPA filters: 14 operating rooms in the hospital, 6 rooms in the Hematopoietic Stem Cell Transplant Unit and the anterooms, 5 rooms in the Endoscopy Unit, 8 cubicles in the Intensive Care Unit (ICU), as well as 2 nursing stations. The controlled environment rooms had a mixed-flow ventilation system equipped with HEPA filters (High-Efficiency Particulate air Filters). These filters efficiently removed 99.97% of airborne particles of 0.3 µm or larger, are replaced every year, and maintenance work on them is performed periodically. The median sizes of the rooms were 45 m² for operating rooms and 30 m² for the rest of the rooms. An exhaustive description of the temperature, the humidity, the seasonality, the periodicity of the measurements performed, the presence of filters, as well as other room characteristics related to the doors, the windows, the surfaces, the presence of patients and medical staff were collected and registered in a systematic way. The presence of patients was registered in 98% of the rooms located in the Hematopoietic Stem Cell Transplant Unit, and in 91% of the ICU rooms. Due to the health condition of these patients, it was decided by the medical staff that performing the corresponding measurements would be taken with patients present. No statistical differences were found between the measurements carried out with or without patients present in the Hematopoietic Stem Cell Transplant Unit and the ICU. In the rest of the rooms studied, all the measurements were carried out with the presence of a maximum number of 3 members: two trained along with a senior staff member who systematically followed the measures concerning hand washing and protective clothing recommended by the CDC.

For the microbiological air samplings, the “Biomérieux Air Ideal” sampler was used, following a volumetric method that, by aspiration, causes an air flow over the culture medium where the microorganisms are attached due to impaction. It takes in 100 liters (L)/min with an impact speed of 20 m/s; the total volume of air taking in was 1000 L. Sabouraud Agar with chloramphenicol was the culture medium to detect fungi, and a Blood Agar was used to identify bacteria.The units of measurement were CFU/m^3^. The counting of total mesophilic aerobic bacteria was carried out after 48 hours of incubation, and the identification and counting of CFU/m^3^ of fungi after a period of five days of incubation.

Following the criteria described in the environmental microbiology study of the Spanish UNE-171340:2012 regulation, fungal contamination was established as the presence of *Aspergillus, Rhizopus, Mucor, or Scedosporium* in the air samples of the room analyzed, and bacterial contamination when the airborne level of bacteria is superior to 100 CFU/m^3^. In order to have only one criteria regarding microbiological contamination, a new indicator was created: Environmental Biocontamination Criterion (EBC). This indicator took into account both fungal and bacterial contamination. EBC was achieved when neither fungal contamination nor bacterial contamination was found. We also decided to create this criterion because it was more restrictive than the criteria described in the previous Spanish regulation.

For the airborne particle counting, the “Handheld LPC Model 3887” laser counter from Kanomax® was used, which measures the concentration of the airborne particles by a photometric system and an air flow of 2.82 L/min. This method allows to differentiate the number and size of particles contained in the air. Each airborne particle has a duration of 5 minutes with a total of 14.1 L of air flow taken in, quantifying the maximum number of particles of 0.3 μm, 0.5 μm, and 5 μm in size by a Light Source Laser Diode. In parallel the microbiological air samplings were also carried out every 5 minutes.

The results of the training period (328 measurements) were analyzed. For the qualitative variables, frequencies and their percentages were used for the descriptive analysis, and the Pearson’s χ^2^ test or Fisher’s exact nonparametric test for their comparison. For the quantitative variables, the mean and their standard deviation (SD) were calculated for the descriptive analysis, and the Student t test or the non-parametric Mann-Whitney U test for their comparison. The area under the ROC curve (aROC) of airborne particle counting and EBC was calculated for the different sizes of particles. The aROC is a graphic representation of sensitivity against 1-specificity. Sensitivity measures the proportion of actual positives that are correctly identified as such, while specificity measures the proportion of actual negatives that are correctly identified as such. The cut-off points that optimize the combination of sensitivity and specificity in the association between airborne particle counting and EBC, using the Youden’s J statistic, were also calculated. The Youden’s J statistic is equal to sensitivity + specificity-1.

Finally, according to Hanley and McNeil methods^15^, it was verified that there were no statistically significant differences between the aROCs of the training period compared to those obtained in the testing period (when 301 measurements were carried out). Moreover, the cut-off points selected for EBC in each of the particle sizes in the training period were validated with the measurements obtained for each of the particle sizes in the testing period, calculating new sensitivities, specificities, predictive values, likelihood ratios, and proportion of correct diagnoses.

Statistical significance was set at a two-sided p < 0.05. The analyses were conducted with SPSS version 24 (IBM, Armonk, NY).

## Results

A total of 629 measurements were carried out, 328 in the training period, and 301 during the testing period. The comparison between the characteristics of the measurements performed during the training and the testing period are presented in Table 1. No statistical significant differences were found when comparing temperature, humidity, bacterial or fungal contamination, or different particle sizes between periods.

**Table 1:** Comparison between the characteristics of the measurements performed during the training and the testing period.

	MEASUREMENTS			
	Total (629)	Training period (328)	Testing period (301)	
	N (%)	n (%)	n (%)	P - value
**Controlled environment rooms**				
Endoscopy Unit	44 (7.0)	24 (7.3)	20 (6.6)	
Hematopoietic Stem Cell Transplant Unit	126 (20.0)	76 (23.2)	50 (16.6)	
Operating rooms	350 (55.6)	162 (49.4)	188 (62.5)	
Intensive Care Unit	109 (17.3)	66 (20.1)	43 (14.3)	
**Bacterial Contamination**				0.345
Yes	52 (8.3)	29 (8.8)	23 (7.6)	
No	577 (91.7)	299 (91.2)	278 (92.4)	
**Fungal Contamination**				0.147
Yes	57 (9.1)	34 (10.4)	23 (7.6)	
No	572 (90.9)	294 (89.6)	278 (92.4)	
**EBC**				0.117
Yes	98 (15.6)	57 (17.4)	41 (13.6)	
No	531 (84.4)	271 (82.6)	260 (86.4)	
	**Mean (SD)**	**Mean (SD)**	**Mean (SD)**	**P - value**
**Airborne particle counting sizes**				
0.3 μm	12413 (31772)	11582 (17252)	13319 (42274)	0.494
0.5 μm	2832 (6069)	2842 (4733)	2820 (7258)	0.965
5 μm	169 (254)	199 (298)	137 (190)	0.002
**Climatic conditions**				
Temperature	22 (3.2)	22 (3.9)	22 (2.5)	0.356
Relative humidity	43 (41.9)	41 (36.3)	44 (46.2)	0.455

SD: Standard deviation. EBC: Environmental Biosafety Criterion. Environmental biocontamination criterion was defined as the absence of both fungal and bacterial contamination.

The overall EBC percentage reached 85% in controlled environment rooms with HEPA filters (p < 0.05). In 97% of measurements carried out in operating rooms, EBC was achieved; 76% in the Hematopoietic Stem Cell Transplant Unit and in the anterooms, and 53% in the 8 cubicles of the Intensive Care Unit (ICU), as well as at the 2 nursing stations.

The comparison between the EBC compliance in the measurements performed during both the training and the testing period are presented in Table 2.

**Table 2:** Comparison between the EBC compliance in the measurements performed during both, the training and the testing period.

	MEASUREMENTS		
	EBC	NO EBC	
	N (%)	N (%)	P-value
**Controlled environment rooms**			<0.001
Endoscopy Unit	17 (38.6)	27 (61.4)	
Hematopoietic Stem Cell Transplant Unit	29 (23.0)	97 (77.0)	
Operating rooms	11 (3.1)	339 (96.9)	
Intensive Care Unit	41 (37.6)	68 (62.4)	
	**Mean (SD)**	**Mean (SD)**	**P-value**
**Airborne particle counting sizes**			
0.3 μm	29817 (39245)	9201 (29118)	<0.001
0.5 μm	7406 (8831)	1987 (4978)	<0.001
5 μm	422 (472)	123 (147)	<0.001
**Climatic conditions**			
Temperature	24 (2.3)	22 (3.3)	<0.001
Relative humidity	58 (92)	40 (21)	<0.001

EBC: Environmental Biosafety Criterion. Environmental biocontamination criterion was defined as the absence of both fungal and bacterial contamination.

Regarding environmental bacterial contamination, 8% of the rooms presented more than 100 CFU/m^3^ of bacteria (0.3% in operating rooms, 13% in Hematopoietic Stem Cell Transplant Unit, 28% in UCI, and 11% in Endoscopy Unit). Environmental fungal contamination was present in 19% of the rooms (2.9% in operating rooms, 13% in Hematopoietic Stem Cell Transplant Unit, 17% in UCI, and 30% in Endoscopy Unit). This fungal contamination consists of *Aspergillus, Rhizopus, Mucor, or Scedosporium*.

**Figure 1:**
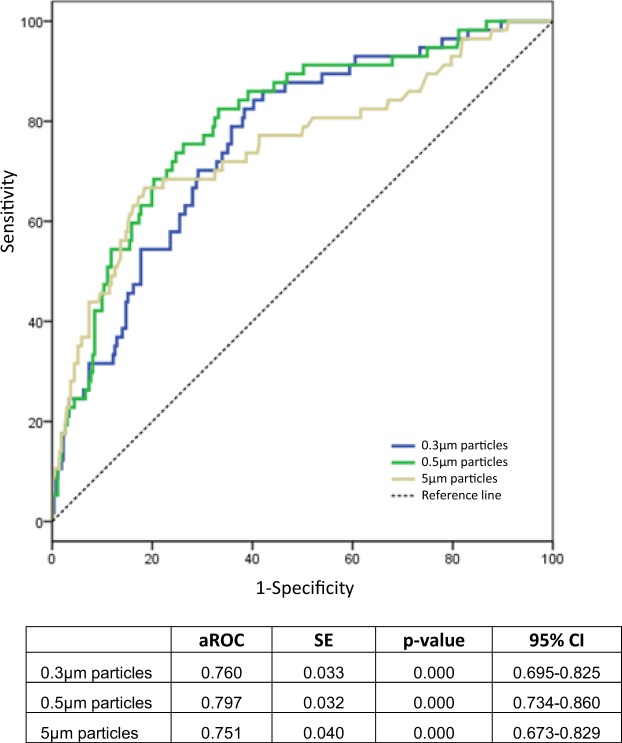
aROCs corresponding to the association between airborne particle counting and EBC in the training period. aROC: area under the ROC curve.

An association was found between airborne particle counting and EBC in the training period, and the magnitude was assessed with aROCs for the different sizes of particles. For 0.3 µm particles, an aROC = 0.760 was observed, 95% Confidence Interval (95% CI) 0.695–0.825; for 0.5 µm particles, we found an aROC = 0.797 (95% CI 0.734–0.860); and for 5 µm particles, an aROC = 0.751 (95% CI 0.673–0.829) was observed. These aROCs corresponding to the association between airborne particle counting and EBC are depicted in Fig. 1.

Using the Youden’s J statistic, the best cut-off points were stablished for each size of particles to identify EBC in the training period. This statistic allows us to choose the best combination of sensibility and specificity. Thus, a counting greater than 9.0 × 10^3^ of 0.3 µm particles correctly diagnoses 72% of the hygiene status of the controlled environment rooms, with a positive likelihood ratio (LR + ) of 2.6; a counting greater than 3.6 × 10^3^ particles of 0.5 µm was able to correctly diagnose 80% of the rooms, with a LR + of 4.1; and a counting greater than 3.2 × 10^2^ particles of 5 µm correctly diagnoses 81% of the rooms, with a LR + of 4.8. This statistic allows us to establish if the probability of there being biocontamination in the air as high.

To verify that the two periods were comparable, (training and testing), the aROC of airborne particle counting and EBC obtained for each size of airborne particle counting (0.3 µm, 0.5 µm, and 5 µm) were compared, and no statistical significance was achieved, except for the 5 µm particles, the ones with the biggest size and less related to microbiological contamination (data not shown).

Finally, with the 301 measurements carried out in the testing period we validated the cut-off points previously established in the training period. Regarding environmental bacterial contamination, 8% of the rooms presented more than 100 CFU/m^3^ of bacteria (0% in operating rooms, 14% in Hematopoietic Stem Cell Transplant Unit, 37% in UCI, and 0% in Endoscopy Unit). Environmental fungal contamination was present in 7.6% of the rooms (0.5% in operating rooms, 16% in Hematopoietic Stem Cell Transplant Unit, 16% in UCI, and 35% in Endoscopy Unit). This fungal contamination consists of *Aspergillus, Rhizopus, Mucor, or Scedosporium*.

The sensitivities, specificities, predictive values, and likelihood ratios of the cut-off points according to the particle sizes are depicted in Table 3. The sensitivities according to the particles sizes in the testing period were 87% (0.3 µm), 73% (0.5 µm), and 50% (5 µm). The corresponding specificities were 69% (0.3 µm), 84% (0.5 µm), and 92% (5 µm).

**Table 3:** Sensitivities, specificities, predictive values, and likelihood ratios of cut-off points according to the particles sizes in the testing period.

Measurements	Sensitivity	Specificity	Positive predictive value	Negative predictive value	Positive likelihood ratio	Negative likelihood ratio
**Particles**	**% (95% CI)**	**% (95% CI)**	**% (95% CI)**	**% (95% CI)**	**% (95% CI)**	**% (95% CI)**
9.0 × 10^3^ of 0.3 µm	87 (75–94)	69 (63–74)	34 (27–42)	97 (93–98)	2.8 (2.3–3.4)	0.2 (0.1–0.4)
3.6 × 10^3^ of 0.5 µm	73 (60–84)	84 (79–87)	45 (35–56)	94 (91–97)	4.5 (3.6–6.0)	0.3 (0.2–0.5)
3.2 × 10^2^ of 5 µm	50 (37–63)	92 (89–95)	56 (41–68)	91 (87–94)	6.5 (4.1–10.2)	0.5 (0.4–0.7)

CI: Confidence Interval.

For 0.3 µm particles, 72% of the rooms were correctly diagnosed; for 0.5 µm particles, 83% of the rooms were correctly diagnosed; and for 5 µm particles, 86% were correctly diagnosed.

## Discussion

The correlation between airborne particle counting and EBC in the controlled environment rooms, obtained an aROC greater than 0.75 for all particle sizes, providing us with an immediate indicator that can help us to take decisions related to biocontamination.

Biocontamination in the air of a controlled environment room compels the hospital to suspend the activity in the room, inspect the air-conditioning system, and conduct a cleaning as well as a terminal disinfection of all the surfaces, For this reason, it is essential to have a tool that can provide a good real time indicator for decision making concerning biocontamination. Our findings concur with those obtained in previous research^16–18^. However, in our study different sizes of airborne particle counting and different hospital controlled environment rooms were included in the analyses, obtaining similar results in all of them, except for the 5 µm particles when we compared the aROCs obtained during the training and the testing period. However, these particles are the ones with the biggest size and are less related to microbiological contamination.

Uriel *et al*. in 2013, carried out a similar study with 144 paired samples of air, 88 in 8 operating rooms with three levels of filtration, and 56 in 9 rooms without air conditioning systems, obtaining a high level of correlation between microbiological results, in fungi as well as in bacteria, and airborne particle counting. The cut-off points were for 0 CFU, 0.015; for >10 CFU, 0.037; and for >100 CFU, 0.053. The sensitivity for these points were 95.83%, 100%, and 100%; and the Kappa Indexes were 0.51, 0.88, and 0.75, respectively^16^. These results were in line with our study results. Nevertheless, neither the different sizes of particles nor the presence of opportunistic filamentous fungi, regardless of the number of CFU, was taking into account in this study.

Other authors have analyzed the association between airborne particle counting in different controlled environment rooms with environmental bacteria, detecting a strong correlation in the majority of the areas analyzed^17,18^. Specifically, the first of these two studies was carried out by Mirhoseini *et al*. in the ICUs, operating rooms, and internal medicine areas in four hospitals in Iran. A total of 80 samples were collected and the average level of bacteria was between 75–1,194 CFU/m^3^ ^17^. Although they did not include the presence of opportunistic filamentous fungi, they took into account the different airborne particles sizes to establish the correlation with the concentration of bacteria. Furthermore, Stocks *et al*., carried out their study during 22 arthroplasty surgeries, finding an average density of airborne particles greater than >500,000 particles/m3 per 10-minute interval and 1,786 CFU were identified (principally gram-positive cocci). With a density of particles of ≥10 µm explained the variation of 41% of CFU density. Both the density of particles and the density of CFU increased with the duration of the surgery and with an increased presence of operating staff^18^.

In the same way, Armadans-Gil *et al*. founda strong correlation between the number of fungi and the airborne particle counting of 0.5 µm and 1 µm inoperating rooms, burn rooms or hematology rooms, as well as harmacy clean rooms^19^. They took 42 simultaneous samples: 24 in operating rooms, 13 in hematology wards, 3 in Pharmacy Service rooms, and 2 in other procedure rooms. The association between the airborne particle counting and the fungal detection was statistically significant for 0.5 µm particles (p = 0.004) and 1 µm particles (p = 0.003).Likewise, Birgand *et al*. performed a large multicenter study with different surgical procedures. In this case, 3 microbiological air counts and 3 airborne particle counts of 0.3 μm, 0.5 μm, and 5 μm particles were carried out in each procedure, concluding that airborne particle counting was associated with an increased number of airborne microbiological counts in operating theatres^20^. These results concur with our findings. Finally, Dai *et al*. also found that the number of biologic particles in the air of operating rooms quantified with a fluorescent particle counting was correlated with the air bacterial counts (Pearson correlation coefficient = 0.76). However, they did not take into account the particles size^21^. On the other hand, Landrin *et al*., found no correlation in a study performed in 2005, but this study was only carried out during a period of 3 months in 4 operating rooms with HEPA filters in which only the concentration of opportunist fungi and airborne particle counting of 0.5 µm were studied^22^. Cristina *et al*. did not find a significant correlation between airborne particle counting and air microbiological counts when particles of a diameter ≥0.5 µm and ≥5 µm were considered in a three-month study in an operating theatre for arthroplasty^23^. However, both studies were carry out over a short period of time and in a small number of operating theatres with HEPA filters, capable of removing particles of a greater size easily. Finally, Wong *et al*. observed that PM 1 and PM 5 did not have an apparent positive correlation with microbial concentration, but on average, PM 5, PM 10 and PM 25 gave small correlation with microbial concentration^24^.

Another important finding of our study is the cut-off points provided in relation to airborne particle counting. Initially, during the training period, the cut-off points were established with high specificities and negative predictive values (80–90%). Secondly, during the testing period, the previously established cut-off points were validated with the new measurements. The results showed that between 72% and 86% of the controlled environment rooms were correctly diagnose depending on the particles sizes, the negative predictive values were elevated for the three types of particles (92–96%), and the positive likelihood ratio were from 2.8 to 6.5. We consider airborne particle counting as a useful, quick, and preliminary measure to identify the presence of biocontamination in controlled environment rooms with HEPA filters. As such, it may be a good real time indicator for initial decision making concerning biocontamination. Subsequently, microbiological results are required to identify biocontamination with certainty.

Another important contribution of this study, is the creation of the EBC concept, which combined the number of CFU of mesophilic bacteria and the identification of opportunistic filamentous fungi, causal agents of invasive fungal diseases. Although some studies took into account the CFU of fungi^16,22^, these studies did not distinguish if the fungi were *Aspergillus, Riphopus, Mucorales, or Scedosporium*. It is important to make this distinction due to the invasive diseases related to them, and more and more frequent in our environment^1^.

A further advantage is the possibility of integrating particle counting with microbiological air samplings to evaluate individual exposure to airborne bacteria, by instantaneous detection of rapid variations that could go undetected by periodic air microbiological counts. It has been also previously concluded by Hamed *et al*. that combining particle counting with bioaerosol sampling could provide prompt information about rapid variations of air quality^25^. Among the limitations, despite the measurements being performed by trained staff in a systematic way, inter-observer differences cannot be rule out. Airborne particle counting was performed with a portable instrument which presumably is less accurate than this of traditional air microbiological sampling methods, affecting the established cut-off points. Some slight variations detected could be due to changes in the background outdoor concentration levels. Although we took into account the temperature, the humidity, the seasonality, the periodicity of the measurements performed, the presence of filters, as well as other room characteristics related to the doors, the windows, the surfaces, and the presence of patients and medical staff, some residual confounding cannot be dismissed. Our results showed no differences in fungal contamination between different seasons, contrary to other study results. In addition, the external validity of our results could be limited due to other variables that could differ between hospitals such as different air-conditioning systems, building structure, and controlled environment rooms in other hospital units.

With these findings we can propose that the regular verification of the biocontamination in the air of controlled environment rooms with HEPA filters should consist of the inspection of the hygiene status, structural inspections, temperature and relative humidity controls, microbiological air sampling, as well as airborne particle counting.

Thus, we consider airborne particle counting as a useful, quick, immediate and preliminary measure to identify the presence of biocontamination in controlled environment rooms. It is necessary to validate these results with further studies carried out in other hospitals, to increase the external validity in order to make recommendations related to airborne particle counting in controlled environment rooms with HEPA filters.
